# Models Predicting Psychosis in Patients With High Clinical Risk: A Systematic Review

**DOI:** 10.3389/fpsyt.2020.00223

**Published:** 2020-03-24

**Authors:** Cristiana Montemagni, Silvio Bellino, Nadja Bracale, Paola Bozzatello, Paola Rocca

**Affiliations:** Department of Neuroscience, School of Medicine, University of Turin, Turin, Italy

**Keywords:** clinical high risk for psychosis (CHR), attenuated psychotic symptoms (APS), brief and limited intermittent psychotic symptoms (BLIPS), genetic risk and deterioration syndrome (GRD), predictive model

## Abstract

**Objective:**

The present study reviews predictive models used to improve prediction of psychosis onset in individuals at clinical high risk for psychosis (CHR), using clinical, biological, neurocognitive, environmental, and combinations of predictors.

**Methods:**

A systematic literature search on PubMed was carried out (from 1998 through 2019) to find all studies that developed or validated a model predicting the transition to psychosis in CHR subjects.

**Results:**

We found 1,406 records. Thirty-eight of them met the inclusion criteria; 11 studies using clinical predictive models, seven studies using biological models, five studies using neurocognitive models, five studies using environmental models, and 18 studies using combinations of predictive models across different domains. While the highest positive predictive value (PPV) in clinical, biological, neurocognitive, and combined predictive models were relatively high (all above 83), the highest PPV across environmental predictive models was modest (63%). Moreover, none of the combined models showed a superiority when compared with more parsimonious models (using only neurocognitive, clinical, biological, or environmental factors).

**Conclusions:**

The use of predictive models may allow high prognostic accuracy for psychosis prediction in CHR individuals. However, only ten studies had performed an internal validation of their models. Among the models with the highest PPVs, only the biological and neurocognitive but not the combined models underwent validation. Further validation of predicted models is needed to ensure external validity.

## Introduction

Psychotic disorders are some of the most serious mental disorders considering the individual and the social impact (1, 2). They represented the 11th cause of disability in the world in 2013 (3). The delay between the diagnosis and the treatment ranges from 1 to 3 years (4) and results in worsening clinical outcomes (5). Therefore, the clinical focus has increasingly shifted to the early detection and treatment with the aim to either attenuate, postpone and globally avoid the transition to psychosis (6), or enhance clinical and functional outcomes of psychosis over time (7, 8). Psychosis does not appear directly in its full-blown form in adults but it gradually develops over time: often the first manifestations already take place in adolescents (9, 10). For most of the patients suffering from schizophrenia and psychotic disorders, the onset of the disease is anticipated by different symptoms: slight changes in belief, thought, and perception that represent mild forms of delusions, formal thought disorder, and hallucinations, respectively (11).

The clinical staging model has been created to catch these progressive changes, with progressively increasing levels of severity over time (12, 13). This model describes psychopathology in a continuum of different subsequent stages. It comprises five different stages, from stage 0 to stage 4, starting from the lowest level of increased risk of mental illness to progressively higher stages of severity, leading to separated but overlapping pathologies at the highest levels (14, 15). Stage 0 includes subjects at increased risk without any kind of symptoms; stage 1 refers to individuals at clinical high risk for psychosis (CHR); stage 2 coincides with the acute phase or crisis, featured by full-blown psychotic symptoms (the full-threshold first episode psychosis), after which an early recovery phase or post- acute phase in the 6–12 months after the onset of the disease occurs; stage 3 encompasses individuals with either persistent illness or recurrent episodes after the first one (12, 13, 16) and stage 4 holds subjects with chronic disease.

This psychopathological model allows to stage this pathology so that different types of interventions, depending on the stage of illness, can be developed. The psychopathology would be more susceptible to intervention strategies in the first phases of the disease and more crystallized and resistant to therapies in the last phases (15).

The CHR criteria include: attenuated psychotic symptoms (APS), representing mild positive symptoms; brief and limited intermittent psychotic symptoms (BLIPS), characterized by transient, non-serious psychotic symptoms lasting part of the day, and lasted for a maximum period of one week after which spontaneously went to remission; and genetic risk and deterioration syndrome (GRD), including patients with family history of psychosis or schizotypal personality disorder, with additional decline in functioning (17).

Frequently, research in the area of psychiatry has as principal focus the transition from CHR to First Episode Psychosis. Help-seeking subjects meeting CHR criteria, regardless of the scale used, have an increased risk to develop psychotic disorders (18), within a period of time that can be considered quite short. According to a meta-analysis storing data from 27 studies including a number of 2,502 patients, 18% of them developed First Episode Psychosis at by 6 months, 22% by 1 year, 29% by 2 years, and 36% by 3 years from initial assessment (19), with about 73% of these developing a schizophrenia spectrum disorder (20).

Overall, compared to the general population, CHR subjects have a 2-year relative risk (RR) to develop psychosis of 460% as compared to general population (29%/0.063%) (21). However, extracting from the overall high-risk entity, its three principal subgroups, patients with BLIPS were at greater risk for developing psychosis (39% vs 19% after 24 months), than patients in the APS and GRD subgroups (22), while the GRD subgroup shows only a slight transition risks of 5% after three years of follow-up (22).

Since most of the studies conducted a follow-up period of not more than 3 years, after this period the transition rate to psychosis is not completely clear. However, most conversions occur during the first year following the evaluation and the conversion rate decreases significantly thereafter, suggesting that the CHR criteria are sensitive to an imminent risk of the onset of full psychosis (23). However, the CHR criteria alone seem to be insufficient in predicting the imminence of the first episode psychosis, given that from 2/3 to 4/5 cases identified through these instruments do not turn into psychosis within a period of 2 years (24). Thus, the aim is to propose a prognostic model that more effectively picks out those individuals who are more likely to switch from ultra-high risk to a first-episode psychosis (FEP) within a given period of time, to adapt treatments to what subjects really need.

Nevertheless, there is not a model of prediction of the transition to psychosis that has been utilized in clinical practice. One explanation can be that psychotic disorders are heterogeneous in phenomenology, pathophysiology, and etiology (25): it means that CHR samples are composed of different and largely heterogeneous subgroups (26). Another reason can be found in the poor quality of the statistical methods used in the studies involved in developing a transition model from CHR stage to full-blown psychosis. A recent review on 91 studies highlighted several shortcomings of this kind of research: poor methods and reporting, no internal or external cross-validation, small sample sizes, and strategies to create these models not well done. Therefore, most of these models probably have overoptimistic and not realistic predictive accuracy (27).

The present study reviews models predicting transition to psychosis, developed to enhance prediction of illness onset in CHR subjects, extending results of a previous study of prognostic accuracy parameters of predictive modeling studies using clinical, biological, neurocognitive, environmental, and combinations of predictors (28).

## Methods

### Literature Search

On January 31, 2019, an electronic search on PubMed was carried out (from 1998 through 2019), using the following search terms: “at risk mental state,” “psychosis risk,” “prodrome,” “prodromal psychosis,” “high risk,” “clinical high risk psychosis”, “attenuated psychotic symptoms”, “APS”, “brief and limited intermittent psychotic symptoms”, “BLIPS”, “brief intermittent psychosis syndrome”, “BIPS”, “genetic risk and deterioration syndrome”, “GRD”, “psychosis prediction,” “psychosis onset,” “predictive model”. The research was restricted to those articles published from 1998 onward, because this is the year in which the first prospective studies with subjects meeting validated CHR criteria have been published (29).

**Figure 1:**
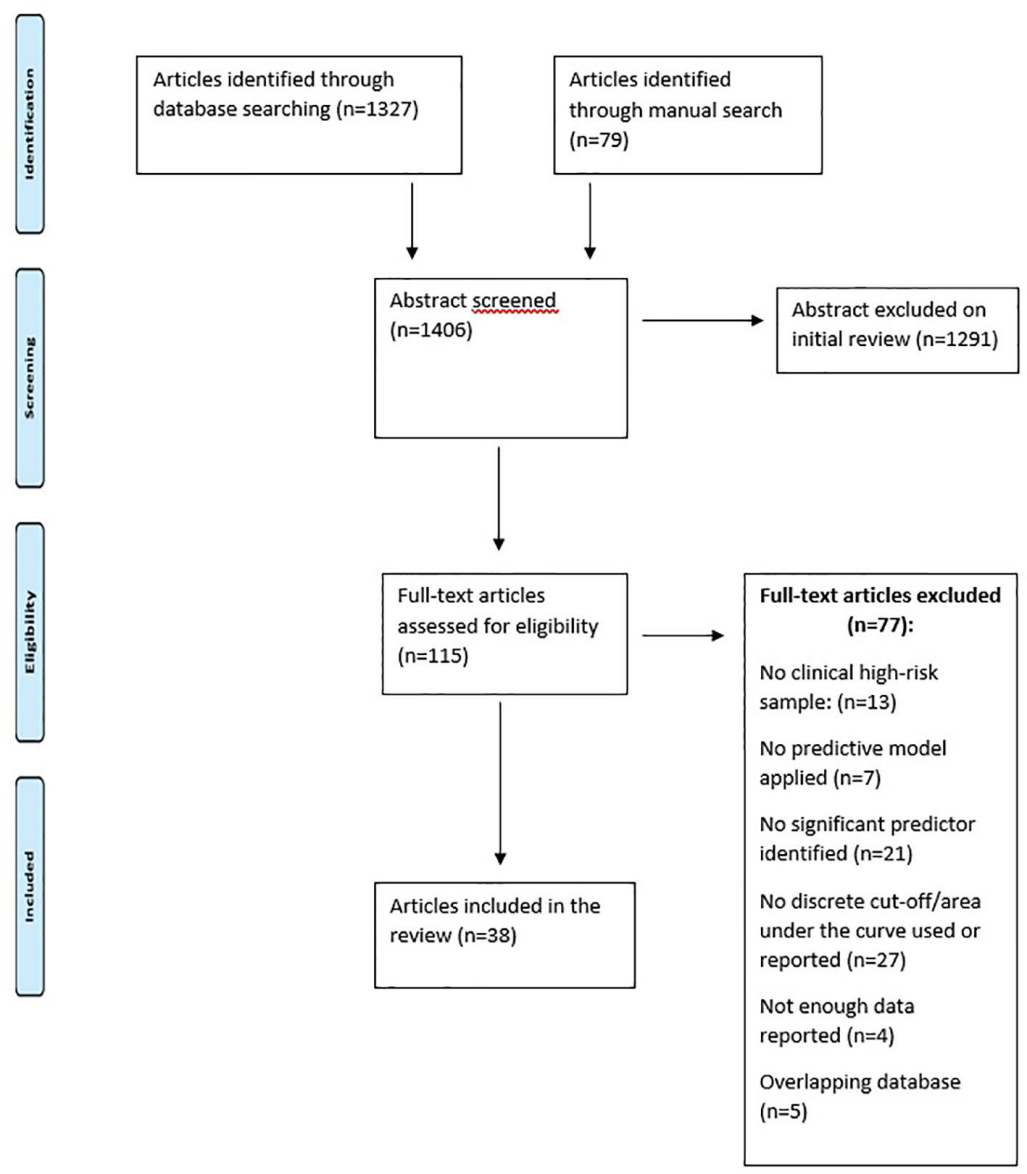
PRISMA flow chart.

This qualitative review was executed according to the Preferred Reporting Items for Systematic Reviews and Meta-Analyses (PRISMA) standard, including evaluation of bias (confounding, overlapping data, publication bias) (30). (**Figure 1**).

Studies were selected in a two-step procedure. First of all, all references retrieved from the databases were screened based on their titles and abstracts. Subsequently, the articles that were potentially eligible were further evaluated based on their full texts. All references within the included studies and those of any previous pertinent reviews were carefully reviewed to identify additional relevant studies. Discrepancies were resolved by mutual discussions. Consensus was then obtained, resulting in a final set of articles that have been reviewed and summarized.

### Inclusion/Exclusion Criteria

As depicted in **Figure 1**, these are the inclusion criteria for the studies in the present review: (a) original articles, to be published in English; (b) presence of CHR subjects [i.e., APS or GRD or BLIPS or brief intermittent psychosis syndrome (BIPS)] according to international standard criteria (6); (c) inclusion of clinical, biological, neurocognitive, environmental, or combinations of predictors to separate CHR individuals who later developed psychosis from those who did not; (d) inclusion of rigorous predictive models, algorithms, or learning systems that predicted later transition to psychosis from variables obtained at baseline, like regression (logistic, Cox proportional hazard model, least absolute shrinkage, and selection operator), support vector machines, or greedy algorithms (31–34).

The following were the exclusion criteria: (a) abstracts, pilot datasets, reviews, articles not written in English; (b) not rigorous statistic methods (i.e., use of mean differences or chi square tests); (c) articles with overlapping datasets using the same predictor. Particularly, when several articles were published using the same population sample, we have chosen the studies reporting the largest sample and most recent data set.

### Recorded Variables

Two investigators (CM, NB) independently realized the extrapolation of data. Several variables have been extracted from the evaluated articles: author, year of publication, demographic characteristics of the CHR sample, predictor domain (clinical, biological, neurocognitive, environmental, combinations), cut-off of predictive variables, type of validation, diagnostic instrument used to define CHR group, administration of antipsychotics, follow-up time, predictive model, and prognostic accuracy data (sensibility SE, specificity SP, positive predictive value PPV, negative predictive value NPV). Moreover, we checked the missing data with all the corresponding authors to record all the information we needed.

## Results

### Selection of Studies

#### Search

**Figure 1** describes the details of what has been searched in literature and the reasons why some articles were excluded. The electronic and manual search described in the previous section provided 1,406 records.

Thirty-eight of these studies met the inclusion criteria: 11 studies made use of clinical predictive models, seven studies used biological models, five studies made use of neurocognitive models, five studies used environmental models, and 18 studies made use of combinations of predictive models across different domains. The results are schematically described in **Table 1**.

**Table 1:** Articles Reporting Predictive Models of Transition to Psychotic Disorder in CHR Subjects.

Articles	Type of CHR diagnostic instrument used	Sample of the CHR subjects (NT/T)	Antipsychotics (patients treated)	Follow-up (months)
Mason et al. (35)	APSS, BPRS, SAPS, SANS	37/37	No	26
Cannon et al. (36)	SIPS	209/82	Yes	30
Nelson et al. (37)	CAARMS, BPRS	197/114	No	60
Nieman et al. (38)	SIPS, BSABS-P	207/37	Yes	18
Bearden et al. (39)	SIPS	33/21	Yes	12
DeVylder et al. (40)	SIPS	74/26	Yes	30
Ziermans et al. (41)	SIPS, BSABS-P	33/10	Yes	72
Riecher-Rössler et al. (42)	BSIP, BPRS, SANS	32/21	No	64
Tarbox et al. (43)	SIPS	192/78	n/a	30
Ruhrmann et al. (44)	SIPS, BSABS-P	146/37	Yes	18
Velthorst et al. (45)	SIPS	119/28	No	24
van Tricht et al. (46)	SIPS	91/22	Yes	18
Perkins et al. (47)	SIPS	40/32	Yes	24
Van Tricht et al. (48)	SIPS, PANSS, PAS	43/18	16*	36
Ramyead et al. (49)	BSIP	35/18	No	36
Koutsouleris et al. (50)	BSIP, BPRS	21/16	4	84
Koutsouleris et al. (51)	BSABS	18/15	No	18
Koutsouleris et al. (52)	BPRS, SANS, PANSS	33/33	No	52
Hoffman et al. (53)	SIPS	19/9	No	24
Koutsouleris et al. (54)	CAARMS, BSABS-P	20/15	No	48
Pukrop et al. (55)	SIPS, BSABS-P	39/44	No	36
Fusar-Poli et al. (56)	CAARMS	129/23	Yes	24
Dragt et al. (57)	SIPS and BSABS-P	53/19	Yes	36
Buchy et al. (58)	SIPS	141/29	No	48
Nieman et al. (59)	SIPS, BSABS-P	43/18	Yes	36
Lencez et al. (60)	SIPS	21/12	No	32
Cornblatt et al. (61)	SIPS	77/15	Yes	36
Michel et al. (62)	SIPS, SPI-A	53/44	Yes	24
Chan et al. (63)	CAARMS	58/18	No	24
Corcoran et al. (64)	SIPS, SOPS	42/7	n/a	24
Gschwandtner et al. (65)	BSIP, BPRS	30/12	No	72
Mittal. et al. (66)	SIPS	66/24	13	24
Rüsch et al. (67)	SIPS	159/13	33	12
Thompson et al. (68)	CAARMS	63/41	No	28
Zimmermann et al. (69)	BPRS, SANS	15/13	4	48
Ruhrmann et al. (44)	BSABS-P, SIPS	208/37	55	18
Yung et al. (70)	CASH, BPRS	68/36	No	12
Yung et al. (71)	CASH, BPRS	29/20	No	12

APSS, the assessment of prodromal and schizotypal symptoms; BPRS, Brief Psychiatric Rating Scale; BSABS-P, The Bonn Scale for the assessment of basic symptoms- prediction list; BSIP, Basel Screening Instrument for Psychosis; CAARMS, comprehensive assessment of at risk mental states; CASH, comprehensive assessment of symptoms and history; CHR, clinical high risk; ERIraos, early recognition inventory based on the retrospective assessment of the onset of schizophrenia; HR, high risk; n/a not available; NT, nontransition; PANSS, Positive and Negative Symptoms Scale; PAS, premorbid assessment scale; PSE, present state examination; SANS; Scale for Assessment of Negative Symptoms; SAPS, Scale for Assessment of Positive Symptoms; SD, standard deviation; SIPS, structured interview for prodromal syndromes; SOPS, Scale of Prodromal Symptoms; SPI-A, Schizophrenia Proneness Instrument, Adult version; T:transition.aData are shown for the CHR subjects with a known outcome (n=183). The total group included 245 subjects.

*16 subjects treated: 9 of them nontransition and 7 transition to psychosis.

For all these studies, validation was evaluated. Some models have internal validation, that means test model in new data, random from underlying population. Other studies have external validation, that means test model in new data, different from development population. Some models have apparent validation, that gives an optimistic estimate of model performance. Some studies have a cross-validation: it means to test the model's ability to predict new data that was not used in estimating it, in order to flag problems like overfitting or selection bias (6) and to give an insight on how the model will generalize to an independent dataset. However, some models do not show any validation.

### Clinical Predictive Models

The 11 studies that have tested the clinical predictive models are described in **Tables 1** and **2**. The clinical parameters included specific positive [odd belief: (35); auditory hallucinations: (35, 45); unusual thought content: (36); illogical thinking: (39); suspiciousness: (36, 42, 43); bizarre thinking: (44); delusions: (45); formal thought disorders: (45); disorganized communication: (40, 41); positive symptoms: (41)], negative [anhedonia/asociality: (35, 43); blunted affect: (35); alogia: (43)] and basic symptoms (41), social and global functioning (35–37, 44), and the Strauss and Carpenter Prognostic Scale (SCPS) (38). In details, we found that the best clinical predictors recognized were schizotypal personality characteristics (35), formal thought disorders (39), specific items of the SCPS assessing quality of useful work and social relations, positive symptoms and subjective distress (38), disorganized communication (particularly, subthreshold thought disorder) both at baseline and as a trajectory of high persistent disorganized communication (40), and early adolescent social dysfunction (43), with baseline prodromal symptoms of disorganized communication, social anhedonia, suspiciousness, and diminished ideational richness that mediate the association with transition to psychosis. We found that several studies presented an increased predictive power when more variables were evaluated together. Particularly, a prediction model was developed and included positive symptoms, bizarre thinking, sleep disturbances, a schizotypal disorder, level of functioning in the past year, and years of education (44). Another study, using the median score of the global assessment of functioning scale (GAF) and the QLS scale, identified a “high” and “low” group (comprising of subjects functioning above or below median at both baseline and follow-up) and a “deterioration” group and “improving” group; Chi-square analyses showed that the low and deteriorating functioning groups were the most likely to develop FEP (45). Otherwise, Cannon et colleagues (36) found that five features contributed uniquely to the prediction of psychosis: a genetic risk for schizophrenia with recent deterioration in functioning, higher levels of unusual thought content, higher levels of suspicion/paranoia, greater social impairment, and a history of substance abuse. Predictive power was increased when prediction algorithms combining two or three of these variables were generated. Other studies have highlighted several different factors associated with transition to psychosis. A study (37) has identified five factors: year of entry into the clinic, duration of symptoms before clinic entry, baseline functioning, negative symptoms, and disorders of thought content. Another study (41) has recognized low IQ, the severity of attenuated positive symptoms, and particularly disorganized symptoms that were identified as highly predictive of functional outcome. A study (42) has identified, as the best transition predictors, selected APS (suspiciousness), negative symptoms (anhedonia/asociality), and cognitive deficits (reduced speed of information processing).

**Table 2:** Prognostic Accuracy Parameters of the Predictive Models Included in the Systematic Review.

Articles	Predictor area		Predictive model	Validation	Predictive variables (Cut-off and/or AUC)	SE (%)	SP (%)	PPV (%)		NPV (%)
Mason et al. (35)	Clinical		Logistic regression	No	Odd belief (SPD ≥ 1), marked impairment in role functioning (APSS ≥ mild), auditory hallucinations (SAPS ≥ 2), anhedonia/asociality (SANS ≥ 2), blunted affect (APSS ≥ mild)	84	86	86		84
Cannon et al. (36)	Clinical		Cox proportional hazard model	No	Unusual thought content (SIPS > 3)	56	62	48		/
					Suspicion/paranoia (SIPS > 2)	79	37	43		/
					Social functioning (SIPS < 7)	80	43	46		/
					Psychosis in first-degree relatives with functional decline (GAF and SIPS)	66	59	52		/
Nelson et al. (37)	Clinical		Cox proportional hazard model	No	Global functioning (GAF < 44), duration symptoms (CAARMS > 738 d)	45	88	72		69
Nieman et al. (38)	Clinical		Cox proportional hazard model	No	SCPS < 49	76	57	24		93
Bearden et al. (39)	Clinical		Logistic regression	No	Illogical thinking score (K-FTDS)	69	71	/		/
DeVylder et al. (40)	Clinical		Cox proportional hazard model	No	Disorganized communication (SIPS > 2, AUC in the 2 through 4 range: 0.64)	81	38	33		85
					Disorganized communication (SIPS > 3, AUC in the 2 through 4 range: 0.64)^b^	62	62	36		82
					Disorganized communication score (SIPS > 4, AUC in the 2 through 4 range: 0.64)	31	81	36		77
Ziermans et al. (41)	Clinical		Logistic regression	No	Positive symptoms (SIPS > 11.5, AUC: 0.80)	40	85	44		/
					Cognitive deficits ≥ 19 (BSABS-P ≥ 19, AUC: 0.79)	67	87	60		91
Riecher-Rössler et al. (42)	Clinical		Logistic regression	No	Suspiciousness (BPRS:0.41, AUC: 0.72)	70	72	61		79
Tarbox et al. (43)	Clinical		Cox proportional hazard model	No	Alogia, anhedonia-asociality (SANS:0.33, AUC: 0.78)	79	68	/		/
					Suspiciousness (SIPS > 3)	53	76	51		75
Ruhrmann et al. (44)	Clinical		Cox proportional hazard model	No	Disorganized communication (SIPS > 1)	72	46	40		76
					Social anhedonia (SIPS >2)	69	58	46		80
					Positive symptoms (SIPS>16), bizarre thinking (SIPS > 2), schizotypal personality disorder (SIPS), highest functioning score in the past year (GAF-M score), sleep disturbances (SIPS>2), years of education, AUC: 0.81	42	98	83		87
Velthorst et al. (45)	Clinical		Logistic regression	Apparent	PANSS, with a score of 4 or more on delusions, hallucinations or formal thought disorder'; having a score of 6 on any of the items of the SIPS-Positive Symptoms subscales for more than 7 d. LCFA to the 19 items of the SIPS.	97.3	86.5	88.3		96.8
Van Tricht et al. (46)	Biological		Cox proportional hazard model	No	Quantitative EEG: occipital-parietal individual alpha peak frequency, frontal delta and theta power.	46	87	56		87
Perkins et al. (47)	Biological		Greedy algorithm	Internal	Blood biomarker: interleukin-1B, GH, KIT ligand, interleukin-8, matrix metalloproteinase-7, interleukin-7, resistin, chemokine [c-c motif] ligand8, immunoglobulin E, coagulation factor VII, TSH, malondialdehyde-modified low-density lipoprotein, apolipoproteinD, uromodulin and cortisol (AUC: 0.88)	60	90	72		84
Van Tricht et al. (48)		Biological	Cox proportional hazard model	No	ERP: P300 (Amplitude < 14.7 microvolt)	83	79		/	/
Ramyead et al. (49)		Biological	LASSO	Internal	Quantitative EEG: lagged phase synchronization, current-source density (AUC: 0.78)	58	83		/	/
Koutsouleris et al. (50)		Biological	Binary SVM with radial basis function	Internal with nested repeated 10-fold cross-validation	MRI-based biomarkers (The neuroanatomical decision functions underlying these results particularly involved the prefrontal perisylvian and subcortical brain structures)	81.0	87.5		77.8	89.5
Koutsouleris et al. (51)		Biological	Binary SVM with radial basis function	Internal with 5-fold cross-validation	Multivariate neuroanatomical pattern classification performed on the structural magnetic resonance imaging data	83	80		83	80
Koutsouleris et al. (52)		Biological	SVM	Internal	Gray matter volume reduction (dorsomedial, ventromedial, and orbitofrontal areas extending to the cingulate and right intra- and perisylvian structures	76	85		83	78
Hoffman et al. (53)		Neurocognitive	Cox proportional hazard model	No	Length of speech illusion (babble task ≥ 4)	89	90		80	94
Koutsouleris et al. (54)		Neurocognitive	SVM	Internal	Verbal and executive functioning (MWT-B, DST, TMT-B, RAVLT-DR, and RAVLT-Ret)	75	80		83	71
Riecher-Rössler et al. (43)		Neurocognitive	Logistic regression	No	Verbal IQ and attention (MWT/TAP Go/NoGo false alarm: 0.38, AUC: 0.62)	80	59		57	83
Pukrop et al. (55)		Neurocognitive	Logistic regression	No	Verbal memory–delayed recall (Auditory Verbal Learning Test), verbal IQ (Multiple Choice Vocabulary Test), verbal memory–immediate recall (Auditory Verbal Learning Test) and processing speed (DST)	75	79		80	74
Ziermans et al. (41)		Neurocognitive	Logistic regression	No	IQ (Wechsler Intelligence Scales < 86.5, AUC: 0.77)	40	97		80	84
Fusar-Poli et al. (56)		Environmental	Log-rank test	No	Unemployment (“yes/no” assessed with unstandardized questionnaire)	57	61		20	89
Dragt et al. (57)		Environmental	Cox proportional hazard model	No	Urbanicity (BDF, ≤100 000 inhabitants), impaired	63	88		63	88
					social-sexual aspects, age 12–15 (PAS), impaired					
					social-personal adjustment, general (PAS)					
Tarbox et al. (43)		Environmental	Cox proportional hazard	No	Early adolescent social maladjustment (PAS > 2)	50	71		46	72
Buchy et al. (58)		Environmental	Cox proportional hazard	No	Alcohol use (“yes/no” AUS/DUS)	69	81		26	90
Cannon et al. (36)		Environmental	Cox proportional hazard model	No	Abuse of alcohol, hypnotics, cannabis, amphetamines, opiates, cocaine, hallucinogens (“yes/no” as assessed by the Structured Clinical Interview for DSM-IV or the Schedule for Affective Disorders and Schizophrenia for School-Age Children)	29	83		43	/
Ziermans et al. (41)		Combination	Logistic regression	No	Positive symptoms (SIPS > 11.5) and IQ (Wechsler Intelligence Scales ≤ 86.5) (AUC: 0.82)	50	91		63	86
Riecher-Rössler et al. (42)		Combination	Logistic regression	Internal	Suspiciousness (BPRS), anhedonia-asociality (SANS) and attention (TAP Go/NoGo false alarm) (cut-off: 0.41, AUC: 0.87)	83	79		71	86
Nieman et al. (59)		Combination	Cox proportional hazard	Internal	P300 amplitude (ERP), social-personal adjustment	78	88		74	90
			model		(PAS) (AUC: 0.86)					
Lencz et al. (60)		Combination	Logistic regression	No	Verbal memory (Wechsler Memory Scale) and positive symptoms (SIPS) (AUC: 0.43)	82	79		69	88
Tarbox et al. (43)		Combination	Cox proportional hazard model	No	Early adolescent social maladjustment (PAS > 2), suspiciousness (SIPS > 3)	28	92		59	70
					Early adolescent social maladjustment (PAS > 2), disorganized communication (SIPS > 1)	42	82		51	72
					Early adolescent social maladjustment (PAS > 2), social anhedonia (SIPS > 2)	43	78		49	72
					Early adolescent social maladjustment (PAS > 2), ideational richness (SIPS > 0)	32	85		50	70
Cornblatt et al. (61)		Combination	Cox proportional hazard model	No	Disorganized communication (SIPS > 2), suspiciousness (SIPS = 5), verbal memory deficit 2 SD below normal, declining social functioning (Global Functioning: Social scale) (AUC: 0.92)	60	97		82	93
Cannon et al. (36)		Combination	Cox proportional hazard model	No	Psychosis in first-degree relatives with functional decline (SIPS and GAF), unusual thought content (SIPS > 3), social functioning (SIPS < 7)	30	90		81	/
Michel et al. (62)		Combination	Cox proportional hazard	Internal	UHR criteria (SIPS), DST deficit t-score < 40, COGDIS criteria (BSABS-P)	57	66		58	65
Chan et al. (63)		Combination	LASSO	10-fold cross validation	22-Analyte panel, CAARMS-positive subscale (AUC:0.90)	89	79		57	96
Corcoran et al. (64)		Combination	Logistic regression	Apparent	Facial emotion discrimination (EMODIFF), Facial emotion recognition (ER40), Negative symptoms (AUC:0.99)	86	98		86	98
Gschwandtner et al. (65)		Combination	Logistic regression model	No	EEG and general psychopathology (SANS and BPRS) (AUC=0.81)	82	73		/	/
Mittal et al. (66)		Combination	Linear discriminant analysis	Internal with leave one out cross-validation	Movement abnormalities (Dyskinesia Identification System: Condensed User), functional domains (WAIS-III, WISC-III), Neurocognition (FSIQ, vocabulary, matrix reasoning, block design, Logical memory I, Logical Memory II	76.0	60		86.3	43
Rusch et al. (67)		Combination	Logistic regression and cox proportional hazard model	Apparent	Positive and Negative symptoms (PANSS), perceived stigma-related harm (validated 8-item self-report measure based on Lazarus and Folkman's (1984) conceptualization of stress appraisal processes; using the median of as a cut off)	58	98		/	/
Thompson et al. (68)		Combination	Cox proportional hazard model	Apparent	Genetic risk with functional decline; high unusual thought content score (>3 on the SIPS); high suspicion/paranoia score (>2 on the SIPS); low social functioning (<7 on the Social Functioning Scale) and history of substance abuse.	37.3	87.2		65.4	68.2
Zimmermann et al. (69)		Combination	Logistic regression	Apparent	Negative symptom scale (SANS) and EEG spectral data (EEG power in seven bands: delta, theta, alpha1, alpha2, beta1, beta2, beta3)	92	87		86	93
Ruhrmann et al. (44)		Combination	Cox proportion hazard model	Apparent	SIPS-Positive score, bizarre thinking, sleep disturbances, schizotypal personality disorder (according to SIPS) highest GAF-M score in the past year, and years of education (AUC: 80.8)	41.7	97.9		83.3	87.0
Yung et al. (70)		Combination	Cox proportional hazard model	Apparent	Belonging to both the Trait and Attenuated Groups, Duration>5 years, SANS attention>2, GAF<40	60.0	92.6		80.8	81.8
Yung et al. (71)		Combination	Cox proportional hazard model	Apparent	Duration of symptoms > 900 d, GAF score < 51, BPRS total > 15, BPRS psychotic subscale > 2, SANS attention score > 1 and HRSD > 18	86	91		80	94

CAARMS, Comprehensive Assessment of At-Risk Mental State; LCFA, Latent Class Factor Analysis; NPV, negative predictive value; PPV, positive predictive value; SE, sensibility; SP, specificity.

Adapted by Schmidt et al. (28).

APSS, the assessment of prodromal and schizotypal symptoms; AUC, area under the curve; AUS/DUS, The Alcohol and Drug Use Scale; BDF, basic data form; BPRS,

Brief Psychiatric Rating Scale; BSABS-P, The Bonn Scale for the assessment of basic symptoms-prediction list; CAARMS, comprehensive assessment of at risk mental states; CODGIS, cognitive disturbances; DST, digit symbol test; EEG, electroencephalogram; ERP, event-related potentials; GAF: global assessment of functioning; HRSD, Hamilton Rating; Scale for Depression; K-FTDS, Kiddie-Formal Thought Disorder Scale; LASSO, least absolute shrinkage and selection operator; MWT, Mehrfachwahl- Wortschatz test; NPV, negative predictive value; PAS, Premorbid Adjustment Scale; PPV, predictive positive value; RAVLT-DR, Rey Auditory Verbal Learning-delayed recall;

RAVLT-Ret, Rey Auditory Verbal Learning-retention; SANS, Scale for Assessment of Negative Symptoms; SCPS, Strauss and Carpenter Prognostic Scale, score; SD, standard deviation; SE, sensitivity; SFS, social functioning scale; SP, specificity; SPD, Schizotypal Personality Disorder subscale of the International Personality Disorder Examination; SIPS, structured interview for prodromal syndromes; SVM, support vector machine; TAP, Testbatterie zur Aufmerksamkeitspr̈fung; TMT, trail-making test; WISC-III, Wechsler Intelligence Scales for Children 3rd ed. for participants ages 11 to 15; WAIS-III, Wechsler Adult Intelligence Scales, 3rd ed; FSIQ, Full Scale Intelligence Quotient; HRSD, Hamilton Rating Scale for Depression.

a Cut-off scores for determining sensitivity, specificity, and accuracy values were derived from the receiver operating characteristic curve.

b The Youden Index (maximal value for sensitivity + specificity − 1) was 0.24 with the optimal cut point of a score of 3 for baseline disorganized communication.

c This model included 58 (of 61) CHR subjects.

The highest PPV of 88.3% was obtained using a model that included measures of delusions, hallucinations or formal thought disorder (45). This model reached a SE of 97.3%, SP of 86.5%, and NPV of 96.8%. The worst PPV (24%) was produced by combining the following items of the SCPS for transition to a first psychotic episode in subjects clinically at high risk (CHR) of psychosis: most usual quality of useful work in the past year, quality of social relations, presence of thought disorder, delusions or hallucinations in the past year, and reported severity of subjective distress in past month, a predictive model that revealed an SE value of 76%, SP of 57%, and NPV of 93% (38).

Validation was not obtained in any clinical predictive model.

### Biological Predictive Models

Seven studies have evaluated the prognostic accuracy of biological predictive models (**Table 2**). These studies have taken into consideration the MRI based biomarkers (50, 52), multivariate neuroanatomical pattern (51), electrophysiological indicators [quantitative EEG: (46, 49); ERP: event-related potentials: (48)], and blood analyses (47). In details, two studies took into consideration quantitative EEG (46, 49). Van Tricht and colleagues (46) determined quantitative EEG (QEEG) spectral power and alpha peak frequencies (APF), founding that power in theta and delta ranges and occipital–parietal APF contribute to the short-term prediction of psychosis and enable a further stratification of risk in CHR samples. Ramyead et al. (49) assessed the individualized prediction of psychosis by detecting specific patterns of beta and gamma oscillations using machine-learning algorithms, determining that transition to psychosis could be predicted from current-source density (CSD). This study found that left superior temporal gyrus, the left inferior parietal lobule, and the precuneus most strongly contributed to the prediction of psychosis, suggesting that CSD measurements extracted from clinical resting state EEG can be useful to improve the prediction to psychosis. A study (47) took into consideration blood biomarkers, measuring expression of plasma analytes reflecting inflammation, oxidative stress, hormones, and metabolism. A “greedy algorithm” selected analytes that best distinguished individuals with clinical high-risk symptoms who developed psychosis. The classifier included 15 analytes (selected from 117). These results support the hypothesis that inflammation, oxidative stress, and dysregulation of hypothalamic-pituitary axes may be prominent in the earliest stages of psychosis and could lead to develop a multiplex blood assay with a potential for high clinical utility. A study (48) analyzed abnormalities on neuroimaging and neuropsychological examinations before the onset of a first psychotic episode, founding that reduced P3 amplitudes (a scalp-recorded late ERP, occurring approximately 300 ms after an attended unusual or task-relevant stimulus.) were identified as the best predictor for subsequent psychosis in the UHR group. The P3 reduction was related to increased social anhedonia and withdrawal and a lower global assessment of social functioning and social personal adjustment. Different studies (50, 52) concentrated their efforts in individuate MRI biomarkers: the first study (50) found that the neuroanatomical decision functions underlying these results particularly involved the prefrontal perisylvian and subcortical brain structures and the second (52) found that the predictor's decision function involved grey matter volume alterations in prefrontal, perisylvian, and subcortical structures, supporting the idea of the existence of a cross-center neuroanatomical signature of emerging psychosis enabling individualized risk staging across different high-risk populations. Finally, another study (51) developed a multivariate neuroanatomical pattern classification on the structural magnetic resonance imaging data of individuals, in order to help predicting transition to psychosis.

The highest PPV of 83% was reached using the predictive variable of the grey matter volumes (grey matter volume alterations in prefrontal, perisylvian, and subcortical structures), with a SE of 76%, SP of 85%, and NPV of 78% (52). This review has been internally validated. However, the study sample was 66 subjects, constituting a rather small sample. Globally, five of these studies (47, 49–52) were cross validated and two were not (46, 48).

The worst PPV (77.8%) resulted from a predictive model including MRI-based biomarkers. This predictive model yielded an SE of 81%, SP of 87.5%, and NPV of 89.5% (50).

### Neurocognitive Predictive Models

Five studies have analyzed the prognostic accuracy of cognitive predictive models (**Table 2**). These studies have provided measurements of IQ (41, 42, 55), verbal memory (54, 55), attention (42), speech perception (53), executive functioning (54), and processing speed (54, 55).

One of the studies (41) showed that low IQ was the single neurocognitive parameter that discriminated patients at ultra-high risk converted to psychosis from individuals who did not. The severity of attenuated positive symptoms was the only significant predictor of a transition to psychosis and disorganized symptoms were highly predictive of functional outcome.

Another study (42) showed that best transition predictors were selected APS (suspiciousness), negative symptoms (anhedonia/asociality), and cognitive deﬁcits (reduced speed of information processing). Prediction of transitions could be enhanced by a stronger weighting of certain early symptoms and by inserting neurocognitive tests into a stepwise risk assessment. Therefore, this study uses neurocognition in addition to clinical parameters for predicting transition to psychosis. Hoffman and colleagues (53) highlighted that elevated LSI (length of speech illusion) scores indicated increased risk of transition to psychotic disorders when individual participating to the study were not taking olanzapine. A further study (54) has demonstrated that patients at risk of transition to psychosis could be identified on an individual basis by evaluating neurocognitive test batteries using multivariate pattern recognition. In another study (55) several cognitive domains were identified as indicators of vulnerability to psychosis. In addition, the results of the article suggest that subtle deficits in verbal abilities (working and long-term memory, executive and intellectual functions) and decreased speed of processing may help to predict transition to psychosis.

Considering verbal and executive functioning in the predictive model (neuropsychological functions were assessed with a cross-domain neuropsychological test battery comprising nine standardized tests that evaluated premorbid verbal IQ, processing speed, working memory, verbal learning and memory, executive functions, and verbal fluency), the highest PPV of 83% could be obtained with a value of SE equal to 75%, SP equal to 80%, and NPV equal to 71% (54). This model is the only one that has been validated in this domain, with an internal validation. However, the sample of the study is quite small, resulting in 35 subjects. The worst PPV of 57% was achieved by using a model including verbal IQ and attention (42). This model yielded an SE of 80%, SP of 59%, and NPV of 83% (42).

### Environmental Predictive Models

The prognostic accuracy of environmental predictive models was evaluated in five papers (**Table 2**). These models have taken into consideration substance abuse (36, 58), unemployment (56), urbanity (57), social-sexual aspects (57), and social maladjustments (43, 57).

Two studies analyzed substance abuse (36, 58). Buchy et al. (58) and demonstrated that low use of alcohol contributed to the prediction of psychosis. This study has also highlighted that prediction algorithms including associations of additional baseline variables known to be associated with psychotic transition increase predictive power compared with substance use alone. Cannon et al. (36) found that different features contributed to the prediction of psychosis, including clinical features and a history of substance abuse (alcohol, hypnotics, cannabis, amphetamines, opiates, cocaine, hallucinogens): predictive power was enhanced when prediction algorithms combining two or three of these variables were developed. Tarbox et al. (43) identified that early adolescent social maladjustment and baseline suspiciousness together demonstrated moderate positive predictive power (59%) and high specificity (92.1%) in predicting transition to psychosis. A study (57) has identified urbanicity, social–sexual aspects, and social–personal adjustment as predictors of transition to psychosis.

Another study (56) showed that unemployment at the ﬁrst contact with the prodromal service may be a risk factor for the development of a psychotic episode.

The best predictive model was obtained in a study conducted on 72 subjects, with values of PPV, SE, SP, and NPV of 63%, 63%, 88%, and 88%, respectively (57). Measures of urbanity, social-sexual aspects, and social and personal adjustment were significant predictors (P < .001). The worst PPV of 26% was achieved by using a model evaluating alcohol use (“yes/no”). This model yielded an SE of 69%, SP of 81%, and NPV of 90% (58).

There are no predicting models evaluating environmental factors that have been validated.

### Combinations of Predictive Models

Eighteen studies (36, 41–44, 59–71) have evaluated prognostic accuracy combining different predictive models across domains (**Table 2**).

Some of these studies concentrated their efforts in develop predictive models combining symptomatology and neurophysiology (59, 65, 69). The first study (59) combined different predictive models, suggesting that predicting transition to psychosis could be improved with a model including premorbid adjustment and information-processing variables (specifically parietal P300 amplitude) in a multistep algorithm combining risk detection and stratification. A second study (65) demonstrated that patients who develop psychosis showed signiﬁcantly more pathological EEG abnormalities than subjects who did not, located more frequently in temporal or frontotemporal regions of the brain. The speciﬁcity of the prediction of psychosis could be increased from 59 to 73% by considering EEG pathology in addition to psychopathology alone. Zimmermann and colleagues (69) have shown that SANS score in combination with EEG power in four bands (delta, theta, beta1, and beta2 bands), respectively, predicted transition significantly in 13 individuals with later transition to psychosis.

Other research studied predictive models focusing on symptomatology and functioning (44, 61, 68, 70, 71). A prediction model was developed including positive symptoms, bizarre thinking, sleep disturbances, a schizotypal disorder, level of functioning in the past year, and years of education (44). Another study (61) developed a ﬁnal predictor model, with a positive predictive validity of 81.8%, consisted of four variables: disorganized communication, suspiciousness, verbal memory deﬁcits, and decline in social functioning during follow-up. A study (68) found three variables associated with transition to psychosis: high unusual thought content scores; low functioning; and having genetic risk with functional decline. Using a combination of two out of three of these features, the predictive validity of determining whether an individual develops psychosis was improved, although using this method the probability of a person not developing psychotic disorder is still quite high at 35%. A study (70) yielded a method of psychosis prediction at 12 months, identifying the following as predictors: poor functioning, long duration of symptoms, high levels of depression, and reduced attention. A combination of family history of psychosis, a recent significant decrease in functioning and recent experience of subthreshold psychotic symptoms was also predictive of psychosis. A study (71) developed a strategy for predicting transition to psychosis, within a relatively brief follow-up period (12 months), combining some highly significant predictors of psychosis: long duration of prodromal symptoms, poor functioning at intake, low-grade psychotic symptoms, depression, and disorganization. A study (67) has developed a predictive model focusing on individuals functioning and stigma. Specifically, this study (67) showed that more perceived stigma stress at baseline predicted transition to schizophrenia after adjusting for age, gender, symptoms (positive and negative symptoms), and functioning. Other studies concentrate on cognitive deficits and symptomatology (41, 42, 60, 62, 66). Another study (41) has identified low IQ, the severity of attenuated positive symptoms, and particularly disorganized symptoms as highly predictive of functional outcome. A study (42) has identified as the best transition predictors, selected APS (suspiciousness), negative symptoms (anhedonia/asociality), and cognitive deficits (reduced speed of information processing). A study (60) demonstrated that prodromal patients (with APS) who later developed psychosis had significantly lower verbal memory scores at baseline, suggesting that verbal memory deficits can represent an important risk marker of transition to psychosis, possibly indicating the presence of a prefrontal-hippocampal neurodevelopmental abnormality. A study (62) found that the combination of a processing speed deficit (digit symbol test) and at-risk criteria (APS plus subjective cognitive disturbances) provides an optimized stratified risk assessment to develop psychosis. A research (66) has studied movement abnormalities and cognitive deficits demonstrating that elevated dyskinetic movements in the upper-body region were correlated with deficits in domains of verbal comprehension, perceptual organization, and both immediate and delayed auditory memory. Further, discriminant function analyses indicated that baseline movement abnormalities and neurocognitive deficits significantly classified subjects at risk to develop psychosis (72.3%). Results support a common cortico-striato-pallido-thalamic circuit irregularity, underlying both movement abnormalities and cognitive deficits in individuals at high risk for psychosis.

Another study focused on maladjustment of individuals at high-risk to develop psychosis. Tarbox et al. (43) identified that early adolescent social maladjustment and baseline suspiciousness together demonstrated moderate positive predictive power (59%) and high specificity (92.1%) in predicting transition to psychosis. It uses also as predictor of transition to psychosis the early adolescent social dysfunction. Other research was carried out in the field of substance abuse. Particularly, Cannon et al. (36) found that different features contributed to the prediction of psychosis, including clinical features and a history of substance abuse (alcohol, hypnotics, cannabis, amphetamines, opiates, cocaine, hallucinogens): predictive power was enhanced when prediction algorithms combining two or three of these variables were developed. Another interesting field that has been faced was about predictive models combining biology and symptomatology. In details, a study (63) developed a combined molecular/symptom-based test. The authors described the development of a serum biomarker test for the identiﬁcation of individuals at risk of transition to psychosis based on multiplex immunoassay proﬁling analysis of 957 serum samples, identifying and validating an optimal panel of 26 biomarkers that best discriminated patients and controls. The performance increased further incorporating the CAARMS (Comprehensive Assessment of At-Risk Mental State) positive subscale symptom scores into the model. Finally, attention was laid on emotion recognition. Specifically, a study (64) showed how deficits in emotion recognition significantly identify subjects who develop psychosis. The authors, moreover, demonstrate that the best classification model for schizophrenia onset included both face emotion processing (facial emotion discrimination and recognition) and negative symptoms. The highest PPV (86.3%) was obtained in a study (66) that took into consideration movement abnormalities, functional domains and neuro-cognition, with values of SE of 76%, SP of 60%, and NPV of 43%.

The worst PPV of 49% was achieved using early adolescent social maladjustment and baseline suspiciousness together as the predictive variables, which produced an SE of 43%, SP of 78%, and NPV of 72% (43).

## Discussion

Although the field of risk prediction in mental health lags behind other areas of medicine, some promising studies have been conducted to begin to ascertain the operative combinations of risk factors for a number of psychiatric disorders (72). These models must be successfully replicated and validated in multiple samples, external to the one used for the model development phase. This often takes many years to be achieved. The use of risk prediction models must be thoroughly evidence based, with research demonstrating that the model is reliable and applicable to the intended populations of individuals (73).

The prediction and prevention of psychotic disorders should include a two-step approach: one step aimed at the identification of individuals in CHR phase, the other aimed to further stratify risk so that “indicated preventive interventions” can be given to patients in the highest risk stratum in an even more targeted and intensive way.

The present review wants to extend the results of a recent review of Schmidt et al. (28). Our review evaluated a total of 38 studies, encompassing clinical, biological, neurocognitive, environmental, or combinations of predictive models from various domains.

Four main findings should be highlighted.

First, while the highest PPVs in clinical (35), biological (51, 52), neurocognitive (54), and combined (66) predictive models were quite high (all above 83), the highest PPV in environmental predictive models was relatively low (63%) (57). This data could be due to the heterogeneity in the environmental factors included in the studies. Moreover, the examined environmental factors were mostly those that have been related with psychotic disorders, particularly substance abuse, urbanicity, and social maladjustment, so that it is possible that their specificity in detecting transition risk to psychosis of CHR is relatively poor, as outlined by a recent meta-analysis (21).

Moreover, regarding the neurocognition, while many previous studies have suggested that it is an important factor in predicting transition to psychosis, there is significant heterogeneity regarding the specific domains implicated: measurements of IQ, verbal memory, attention, speech perception, executive functioning, and processing speed (74, 75).

Second, none of the combined models showed a superiority when compared with more parsimonious models (using only neurocognitive, clinical, biological, or environmental factors). Thus, based on this data, it could be inferred that a strong PPV can be reached making use of psychopathological or neurocognitive data alone, therefore this approach should be preferred to, i.e., extensive neuroimaging batteries. However, a study (28) estimating the theoretical PPV of a sequential three-stage testing (that contained various combinations of three models predicting transition to psychosis, eg, electroencephalography/clinical, images taken from MRI, and blood indicators) following the initial CHR assessment, has shown that the highest value of PPV was obtained when using in sequence a combined model (clinical + EEG) and two biological models (structural MRI and blood indicators). Particularly, PPV reached a value of 98% for subjects with three positive tests, 71–82% for subjects with two positive complementary tests, 12%–21% for subjects with one positive complementary test, and 1% for subjects without any positive tests. This study could indicate that testing in sequence CHR individuals with models of prediction psychosis onset across multiple domains could substantially enhance psychosis prediction after the initial CHR assessment. Thus, multistage sequential testing enables individual risk stratification of CHR subjects to be made and improve prediction of transition to psychosis. Third, it should be highlighted that only a few studies have tried to replicate directly each other's risk algorithms. Consequently, most published predictive performance estimates are likely to be considerably overoptimistic. Only ten studies have used a strict prognostic accuracy method matching appropriate predictive models provided of internal validation. Some of the studies presented an “apparent validation”, obtained on sample used to develop model, leading to strongly overoptimistic results. The majority part of the studies lacked sufficient details to precisely apply the model in a new dataset and this can be partly explained by the fact that there are no models externally validated.

In order to create rigorous risk prediction models, validation is one of the most important elements. A useful prediction model should give accurate estimates of risk, that can be used from the physicians to help them in clinical management and decision making. Moreover, this model should have a core role in predict individuals' outcome and cost-effectiveness of care. There is a substantial difference between models with internal and external validation. When new individuals were subjected to predictive model provided of internally validation, the performance is was lower than the one observed in the sample used to develop the model (76).

Fourth, our review found that poor conduct and reporting were quite common in both predictor finding and model developed studies. The results of our review highlight that one of the biggest limitations is that most of the studies were based on small sample sizes and number of events (particularly patients with transitions to psychosis) relative to the number of evaluated predictor variables. Small number of evaluated predictor variables ratios enhance the risk of overestimating the performance of the model, if it is developed and assessed in the same sample. When sample sizes are small, as is it frequently occurs in the field of prediction of psychosis research, their performance advantage resulting from the increased ability to capture the true underlying relationship between predictors and response might not be high enough to compensate for the increased risk to overestimate.

Prediction in psychiatry needs to be considered a core aspect for testing hypotheses regarding clinically relevant issues (77). However, there are different problems that have to be faced before develop risk prediction models in psychiatry: one of them is the lack of availability of biological markers of illness; another is the idea that a particular discrimination value (e.g., an AUC threshold of 0.80) is required before clinical adoption. Indeed, in most prediction algorithms, including those regarding the Framingham risk score (FRS), the AUC often ranges from 0.75 to 0.80 (78).

Nevertheless, it's clear that a risk prediction model is useful only if early and preventive intervention are available and effective to prevent individual at high risk in developing disease. The use of validated risk prediction algorithms, despite being available, has delayed in primary care (79). If effective predictive models were designed, all the efforts should be done to make them useful and suitable for clinicians. In fact, quantification of validated prediction model impact in clinical care should be the target to be reached for implementation of these models. Though, impact studies are even less frequently performed than validation studies, as it can be elicited from literature (80).

The research about risk prediction models should progress together with the development of preventive interventions, i.e., long-chain ω-3 polyunsaturated fatty acids (PUFAs). Although ω-3 PUFAs treatment is attractive for prevention from a pathophysiologic perspective, preventive efficacy of ω-3 PUFAs for psychosis had been demonstrated in one single-site *r*andomized, double-blind, placebo controlled trial which has compared ω-3 PUFAs with placebo (81), and later confirmed in a naturalistic, long-term follow-up (82). However, two large replication trials, the NEURAPRO trial (83) and the NAPLS-2 trial (84), did not confirm the hypothesis that ω-3PUFAs may be helpful to prevent psychosis in CHR individuals. The authors have hypothesized that such discrepancies might be explained by different overall transition rates, and by a ceiling effect due to concomitant antidepressant treatment. Other trials are currently underway to this end (Placebo-controlled Trial in Subjects at Ultra-high Risk for Psychosis With Omega-3 Fatty Acids in Europe, PURPOSE, NCT02597439). The efficacy of ω-3PUFAs in sub-groups of patients should also be investigated—for example, in those with aberrant membrane fatty acid levels or inflammatory markers

However, until now, recent meta-analyses have not found robust evidence to favor specific preventive interventions, as confirmed by a recent umbrella review (85), i.e. a review of seven meta-analyses in the field of preventive interventions for psychosis in CHR individuals.

Several methodological limitations of our systematic, qualitative review must be acknowledged. First, we excluded articles published in languages other than English. Second, given the relative scarcity of research on this topic to date, and the variability across studies, we were not able to conduct a quantitative systematic review or meta-analysis. Meta-analytic results would be useful to provide important information regarding common predictors and the predictive power of existing models, but these are infeasible at present given the very limited state of research in this neglected area of clinical psychiatry. Third, combining the three CHR subgroups, populations may confound predictors and have an impact on the overall conversion rates (86) and, therefore, contribute to inconsistencies across sites. As an example, if compared with individuals at genetic high risk, people with intermittent psychosis are more severely impaired and develop more frequently acute psychosis (86). Therefore, extrapolating from the whole high-risk category its three fundamental subgroups, it is likely that more accurate predictors may be detected (61).

In conclusion, our systematic review revealed that poor methods and reporting are very common in prediction of psychosis research. In line with what has been reported above, measures of discrimination and calibration of risk prediction models have been reasonable. Most of the studies are based on small samples, did not perform internal or external cross-validation, and used poor model development strategies, and this is the reason why most published models are probably overestimated and their reported predictive accuracy is likely to be overoptimistic. Therefore, the science of risk prediction models in psychiatry is at the beginning, and this is clearly evident looking at the numerous limitations that these studies revealed. However, research on validation must be done. To make these models useful in clinical practice, predictors must be easily available and assessable, and people at high risk must have access to preventive intervention, that could be considered effective and with a minimal risk of side effects. As such, further research must be conducted to create and improve efficient but also focused preventive interventions. As for psychotic disorders, research is growing up, especially toward the direction of the risk prediction and risk stratification. The research must go forward and our goal must be to make effective prediction and prevention possible.

## Author Contributions

CM and NB contributed to summarize the literature data and to write the review. NB collected literature data and organized the tables. PB, SB, and PR contributed to writing and supervising the review.

## Funding

This study was supported by Ministero dell'Istruzione, dell'Università e della Ricerca-MIUR projects “Dipartimenti di Eccellenza 2018-2022” to the Department of Neuroscience “Rita Levi Montalcini.”

## Conflict of Interest

The authors declare that the research was conducted in the absence of any commercial or financial relationships that could be construed as a potential conflict of interest.
